# Cooperation and conflict: field experiments in Northern Ireland

**DOI:** 10.1098/rspb.2014.1435

**Published:** 2014-10-07

**Authors:** Antonio S. Silva, Ruth Mace

**Affiliations:** Department of Anthropology, University College London, London WC1H 0BW, UK

**Keywords:** evolution of cooperation, parochial altruism, intergroup conflict, real-world measure

## Abstract

The idea that cohesive groups, in which individuals help each other, have a competitive advantage over groups composed of selfish individuals has been widely suggested as an explanation for the evolution of cooperation in humans. Recent theoretical models propose the coevolution of parochial altruism and intergroup conflict, when in-group altruism and out-group hostility contribute to the group's success in these conflicts. However, the few empirical attempts to test this hypothesis do not use natural groups and conflate measures of in-group and unbiased cooperative behaviour. We conducted field experiments based on naturalistic measures of cooperation (school/charity donations and lost letters' returns) with two religious groups with an on-going history of conflict—Catholics and Protestants in Northern Ireland. Conflict was associated with reduced donations to out-group schools and the return of out-group letters, but we found no evidence that it influences in-group cooperation. Rather, socio-economic status was the major determinant of cooperative behaviour. Our study presents a challenge to dominant perspectives on the origins of human cooperation, and has implications for initiatives aiming to promote conflict resolution and social cohesion.

## 1. Introduction

The notion of parochial altruism chimes with our folk belief that group members pull together in times of adversity; for example, during the second World War, the term *Dunkirk spirit* came into common use following the evacuation of Allied troops across the English Channel aided by civilians, at a great risk to themselves, using flotillas of pleasure boats and working barges at the Battle of Dunkirk in 1940 [1]. Recently this idea has been formalized through a series of mathematical models in which intergroup conflict plays a prominent role in the evolution of cooperation. Models of multi-level selection depend on competition between groups over access to resources (such as food, mates or territory) for cultural or genetic traits that harm the individual and favour the group, such as altruism, to be selected [2–4]. In situations of intergroup conflict, it is argued that the combination of in-group altruism and out-group hostility—in what is termed parochial altruism—provides a selective advantage to groups, resulting in the coevolution of parochial altruism and intergroup conflict by group extinction through conquest and assimilation.

Studies in the laboratory and the field have shown an association between cooperative behaviour and intergroup conflict [5–10]. While it should be noted that this type of cooperative behaviour is not necessarily associated with altruism *sensu stricto* (i.e. lifetime fitness costs to the actor), as described in the models of parochial altruism [2–4], the findings from these studies are normally put forward as supporting empirical evidence [6–10]; a study in Burundi found that individuals who suffered the most during the conflict between Hutus and Tutsis were more likely to donate to an anonymous member of their community in a version of a dictator game [7], teenagers (but not children and adults) in Georgia and Sierra Leone were more egalitarian in a sharing game to in-group than to out-group members [9], and senior citizens in Israel were more likely to reject an unfair offer in an ultimatum game during the Israel–Hezbollah war when compared with before and after the war [8].

However, these studies are hindered by methodological limitations that reduce their explanatory power of real-world evolutionary dynamics. First, the majority do not distinguish between different types of cooperative behaviour, conflating in-group with unbiased cooperation (i.e. cooperation with a neutral group), and also failing to measure out-group cooperation (i.e. cooperation with a rival group) [7,8,10]. Yet, the accurate identification of the specific type of cooperative behaviour is crucial in the models of the evolution of cooperation through intergroup conflict, as a group benefit is only obtained if cooperation is aimed towards the in-group and not indiscriminately applied [11]. Second, the experimental set-up of these studies [5–10], while sometimes based in a setting of conflict, never consists of games played between individuals from both groups that are in actual conflict, instead using children from different schools [9], anonymous neighbours who may or may not have shared group membership [7] or senior citizens from the same ethnic group [8]. Experiments using these types of abstract group categorization may not reflect the true dynamics of intergroup competition and prompt the subjects to play according to other real-life cooperative social norms that are not relevant to the hypotheses being tested [12–15]; for example, the Orma of Kenya are more likely to contribute to a public good game, as the game is similar to an existing structure of social contribution, the *harambee* [15,16]. Thus, other groups with lower average contributions are not necessarily less cooperative; it may just be that the games invoke no real-life norm for those groups. Finally, there is evidence of a lack of consistency between different game-based measures of cooperation within the same individuals and populations [12], alongside concerns that players in some traditional economic games may not fully comprehend the pay-off structure involved [17,18].

In our study, we address these issues by establishing an experimental set-up based on real-world institutions and cultural groups, and the use of novel naturalistic experimental methods, school/charity donations and lost letters. Our experimental design aims to capture the context-dependent nature of cooperation by measuring cooperative behaviour in a real-world setting, with the lost letter experiment indicating a time commitment to find a post-box and the donation experiment associated with a monetary cost and benefit. In particular, the use of donations to primary schools in our experiments intends to reflect actual intergroup grievances in Northern Ireland associated with school funding [19]. The individuals in our study are not aware that the donations or lost letters were part of an experiment, therefore minimizing the artificiality typical of most laboratory and field-based economic games.

We measure cooperative behaviour using two groups with a long and ongoing history of conflict: Catholics and Protestants in Belfast, Northern Ireland. This conflict dates back to the seventeenth century, but a renewed bout of violence erupted in the 1960s resulting in over 3000 people killed and tens of thousands injured [20]. The intensity of the conflict has eased since the Good Friday Agreement in 1998, but in 2011 alone over 130 sectarian bombings and shootings were recorded [21]. The levels of residential, marital and educational segregation between the two groups are striking; the large majority of the population still live in segregated neighbourhoods (sometimes separated by separation walls); 94% of children attend segregated schools [21] and only 12% of marriages are between people of a different religion [22]. These two endogamous communities thus have high levels of segregation, a history of violent conflict and clearly defined group boundaries and institutions, making them a highly relevant population in which to test hypotheses related to the evolution and maintenance of cooperation through intergroup conflict.

## 2. Methods

We ran two large-scale experiments—school donations and lost letter experiments—to measure biased (towards the in-group or out-group) and unbiased cooperative behaviour across different Belfast neighbourhoods representing a wide range of socio-economic characteristics.

**Figure 1.**
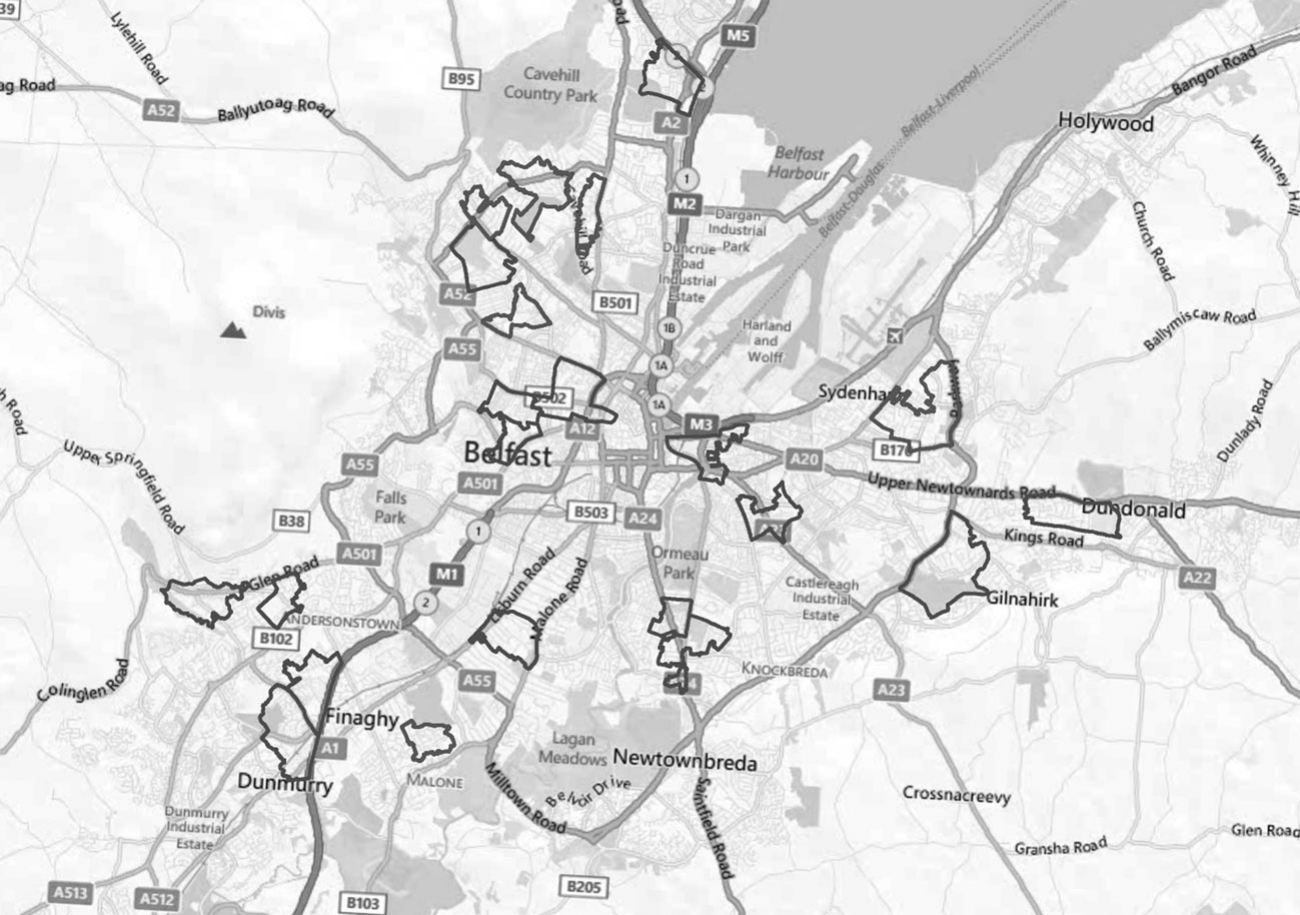
Sample of 22 neighbourhoods in Belfast, UK where the surveys and lost letters experiment were conducted. See the electronic supplementary material, figure S2 for subsample of neighbourhoods where the donations experiment was conducted.

First, we conducted a door-to-door survey of 940 individuals in 22 neighbourhoods (figure 1) in which people received £5 for their participation. The questionnaire included questions on individual socio-economic status (SES) and experiences of the conflict, specifically questions on whether the individual had been attacked or felt threatened by the other group. We created a sectarian threat index from a factor analysis of variables related to the individual exposure to sectarian attacks and threat, which we used as a measure of intergroup conflict (see the electronic supplementary material).

From this survey sample, we randomly allocated a subsample of 466 individuals in 16 neighbourhoods (electronic supplementary material, figure S2) to take part in the donation experiment that was conducted immediately after the completion of the questionnaire. The subsample random allocation was determined by performing the donation experiment at two out every three houses visited in the neighbourhood. Individuals in this subsample were offered the possibility of donating part or all of the money to the local Catholic or Protestant primary school or a neutral charity unaffiliated with any religious group, *Save the Children*. Individuals were only offered the option to donate to a single institution, which was randomly allocated.

Second, we ran a lost letter experiment [23] for which we dropped 832 stamped letters in the same 22 neighbourhoods where the survey was conducted in two rounds in May and June 2012 (*n* = 624) and 2013 (*n* = 208). These stamped letters were addressed to either fictional sectarian or neutral charities (*CatholicAID*, *ProtestantAID* and *CancerAID*; electronic supplementary material, figure S4) and were dropped by two researchers on the pavement with the address facing up on rain-free days. To avoid a return bias dependent on the day and time that the letters were dropped (e.g. when the postman or street cleaners come), each neighbourhood was visited three times at three different time slots (morning, lunchtime and afternoon) on three different days, which were randomly selected (see the electronic supplementary material for more details).

The school donation is a natural experiment that has essentially the same pay-off structure as a dictator game [24], albeit one that is administered surreptitiously and involves real-life cooperative behaviour involving an institution. We are then able to measure the level of cooperation towards a neutral institution (donating to Save the Children), an in-group institution (e.g. Catholic individual donating to a Catholic school) and an out-group institution (e.g. Catholic individual donating to a Protestant school). The lost letter experiment provides an additional measure of cooperative behaviour; we measured unbiased cooperation by the return rate of letters addressed to CancerAID and biased cooperation by the return rate of letters addressed to CatholicAID and ProtestantAID in predominantly Protestant and Catholic neighbourhoods (more than 75% composition of one group), measuring in-group cooperation when the letter is addressed to an organization representing the neighbourhood's majority group and out-group cooperation when the letter is addressed to an organization representing the neighbourhood's minority group.

We test two main hypotheses derived from the theoretical models of intergroup conflict and parochial altruism [2–4]. First, we predict increased exposure to intergroup conflict will be associated with both increased in-group cooperation and decreased out-group cooperation (i.e. parochial altruism), at both the individual and neighbourhood level. Second, we predict intergroup conflict will better explain the variation in in-group cooperation than unbiased cooperation. To test these hypotheses we use multi-level logistic regressions with the binary response variable of donation or no donation and a logistic regression with the binary response variable of the return or not of a lost letter. We ran one regression for overall donations and one for lost letters' overall returns, plus three separate ones by treatment type. The main explanatory variables of interest are the individual level of sectarian threat for the donation analyses and the neighbourhood aggregate level of sectarian threat for the lost letters analyses, and the interactions between these threat variables and the three different treatments (neutral, in-group and out-group). In the donation analyses, we controlled for individual age, gender, educational level household income, religion, having children and neighbourhood level of religious heterogeneity; for the lost letter analyses, we controlled for religious composition, aggregate household income, number of post-boxes, population density and level of religious heterogeneity at the neighbourhood level (see the electronic supplementary material for more detail). The multi-level structure of the analysis allows us to control for the non-independence of individuals' behaviour clustered at the neighbourhood level [25].

## 3. Results

The majority of people chose to donate (68.0%), with 76.6% donating to Save the Children, 76.1% to an in-group school and 51.5% to an out-group school. The majority of lost letters were also returned (61.6%), with 65.3% of CancerAID letters, 62.5% of in-group letters and 53.3% of out-group letters being returned (table 1). We found clear evidence for the existence of parochialism with individuals 25% more likely to donate to an in-group school than an out-group school and 11% more likely to return an in-group letter than an out-group letter (electronic supplementary material, tables S4 and S5).

**Table 1.** Percentage of donations to schools/charity for Catholic and Protestant individuals, and number of lost letters returned by letter and neighbourhood type. (Catholic neighbourhood: >75% Catholic; Protestant neighbourhood: <25% Catholic; Mixed neighbourhood: 25%< >75%.)

donation type	individual					
	overall (*n* = 497)		protestant (*n* = 239)		catholic (*n* = 258)	
overall (*n* = 497)	£0	32.0%	£0	36.8%	£0	27.5%
	£5	62.4%	£5	56.9%	£5	67.4%
	other	5.6%	other	6.3%	other	5.0%
protestant (*n* = 166)	£0	34.4%	£0	25.3%	£0	42.5%
	£5	59.0%	£5	68.4%	£5	50.6%
	other	6.6%	other	*6*.*3%*	other	6.9%
catholic (*n* = 164)	£0	38.4%	£0	55.0%	£0	22.6%
	£5	56.1%	£5	38.8%	£5	72.6%
	other	5.5%	other	6.2%	other	4.8%
neutral (*n* = 167)	£0	23.4%	£0	30.0%	£0	17.2%
	£5	71.9%	£5	63.8%	£5	79.3%
	other	4.7%	other	6.2%	other	3.5%

	neighbourhood					
letter type	overall (*n* = 1056)	protestant (*n* = 384)	mixed (*n* = 336)	catholic (*n* = 336)		
overall (*n* = 1056)	61.6%	61.5%	65.2%	58.0%		
protestant (*n* = 352)	59.9%	64.1%	64.3%	50.9%		
catholic (*n* = 352)	59.4%	55.5%	62.5%	60.7%		
neutral (*n* = 352)	65.3%	64.8%	68.8%	62.5%		

**Figure 2.**
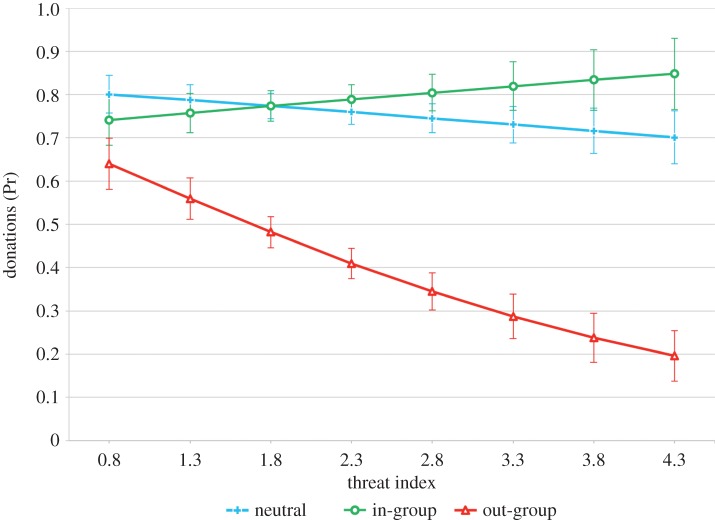
Donations by individual threat index. Predicted probability of an individual donating to an in-group, out-group and neutral institution by the level of individual threat index. This measure is a continuous factor composed of the variables related to the individual exposure to sectarian attacks and threat (details are provided in the electronic supplementary material). This effect is controlled for individual age, gender, educational level, household income, religion, having children and neighbourhood level of religious heterogeneity. Error bars represent the standard errors. (Online version in colour.)

**Figure 3.**
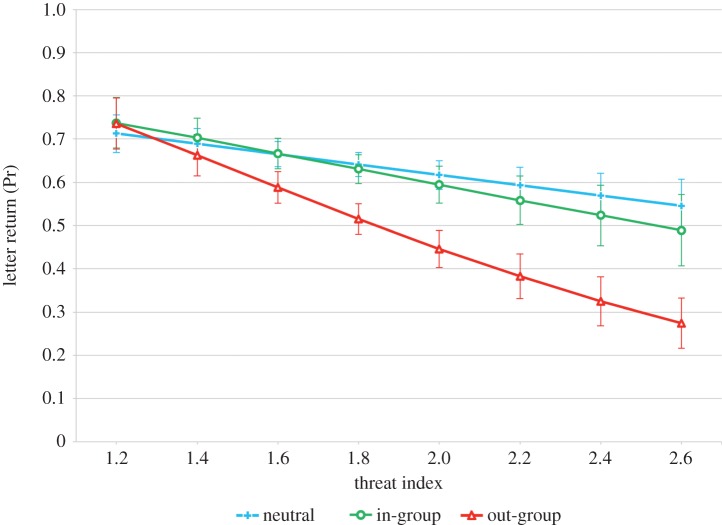
Letters returned by neighbourhood threat index. Predicted probability of return of in-group, out-group and neutral lost letters by the level of neighbourhood threat index. This measure is the neighbourhood aggregate of the continuous factors, which are composed of the variables related to the individual exposure to sectarian attacks and threat (details are provided in the electronic supplementary material). This effect is controlled for neighbourhood religious composition, income deprivation, number of post-boxes, population density and level of religious heterogeneity. Error bars represent the standard errors. (Online version in colour.)

We also found that intergroup conflict was associated with reduced levels of out-group cooperation; individuals who had experienced greater sectarian violence and felt the most threatened by the other group were less likely to donate money to an out-group school (table 2), and in neighbourhoods with higher mean threat levels a lost letter addressed to an out-group institution (relative to the majority population) was less likely to be returned (table 3). At the mean values for all other traits, individuals with the lowest threat levels had a 64% chance of donating to an out-group school, compared with 20% chance for individuals with the highest threat levels (figure 2). For lost letters there was a 70% chance of out-group letters being returned in low threat neighbourhoods, compared with only 30% in high threat neighbourhoods (figure 3). However, we found no evidence for an association between intergroup conflict and cooperation with the in-group, with neither individual nor neighbourhood threat levels significantly predicting donations to in-group schools or returns of in-group letters, respectively (tables 2 and 3; figures 2 and 3). We also did not find any evidence that proximity between groups—measured by the levels of religious heterogeneity in a neighbourhood—predicts any type of cooperative behaviour in either donations or lost letters (tables 2 and 3).

**Table 2.** Incidence rate ratios and 95% confidence intervals from multi-level logistic regressions used to predict overall donations (*n* = 466), and neutral (*n* = 158), in-group (*n* = 153) and out-group donations (*n* = 155; Save the Children, Catholic or Protestant primary schools). (**p < 0.01; *p < 0.05.)

donations variable	overall IRR [95 CI]	neutral IRR [95 CI]	in-group IRR [95 CI]	out-group IRR [95 CI]
out-group donation (ref. in-group)	1.12 [0.46;2.76]	—	—	—
neutral donation (ref. in-group)	1.37 [0.62;3.02]			
threat index	1.1 [0.80;1.53]	0.94 [0.68;1.28]	1.13 [0.77;1.67]	0.63 [0.40;1.00]*
threat index × out-group donation (ref. threat index × in-group donation)	0.58 [0.35;0.96]*	—	—	—
threat index × neutral donation (ref. threat index × in-group donation)	0.82 [0.54;1.24]	—	—	—
GCSE (ref. primary school)	1.33 [0.92;1.93]	1.39 [0.75;2.58]	1.21 [0.62;2.34]	2.07 [0.94;4.56]
A-level (ref. primary school)	1.58 [1.03;2.43]*	1.31 [0.69;2.50]	1.63 [0.59;4.49]	3.13 [1.27;7.72]*
undergraduate (ref. primary school)	1.41 [0.88;2.26]	2.57 [1.05;6.31]*	0.92 [0.4;2.13]	1.91 [0.68;5.40]
graduate (ref. primary school)	2.02 [1.11;3.68]*	1.72 [0.61;4.86]	1.13 [0.31;4.1]	6.59 [1.94;22.35]**
mid HH income (ref. low HH income)	1.38 [0.99;1.94]	1.53 [0.88;2.68]	1.51 [0.82;2.79]	1.28 [0.67;2.46]
high HH Income (ref. low HH income)	1.81 [1.21;2.70]**	1.61 [0.83;3.14]	3.45 [1.44;8.28]**	1.09 [0.47;2.48]
male (ref. female)	1.09 [0.84;1.42]	1.20 [0.73;1.95]	1.38 [0.86;2.23]	0.72 [0.42;1.22]
age	1.01 [1.00;1.02]*	1.01 [0.99;1.03]	1.02 [1.00;1.04]*	1.01 [0.99;1.03]
protestant (ref. Catholic)	0.76 [0.58;1.01]	0.72 [0.45;1.15]	0.9 [0.56;1.47]	0.69 [0.40;1.18]
children (ref. no children)	1.62 [1.17;2.25]**	2.00 [1.12;3.54]*	2.06 [1.09;3.90]*	0.84 [0.46;1.52]
religious heterogeneity	1.00 [0.99;1.01]	1.00 [0.99;1.02]	1.01 [0.99;1.02]	1.00 [0.98;1.01]

**Table 3.** Incidence rate ratios and 95% confidence intervals from logistic regressions used to predict the return of all lost letters (*n* = 832), neutral (*n* = 352), in-group (*n* = 240) and out-group (*n* = 240) lost letters (addressed to CancerAID, CatholicAID or ProtestantAID). (**p < 0.01; *p < 0.05.)

lost letters variable	overall IRR [95 CI]	neutral IRR [95 CI]	in-group IRR [95 CI]	out-group IRR [95 CI]
in-group donation (ref. neutral)	1.40 [0.44;4.39]	—	—	—
out-group donation (ref. neutral)	2.72 [0.82;9.1]	—	—	—
threat index	0.90 [0.55;1.49]	0.87 [0.45;1.68]	0.41 [0.13;1.29]	0.20 [0.07;0.63]**
threat index × in-group donation (ref. threat index × neutral donation)	0.81 [0.43;1.55]	—	—	—
threat index × out-group donation (ref. threat index × neutral donation)	0.47 [0.23;0.94]*	—	—	—
mid HH income (ref. low HH income)	1.69 [1.25;2.3]**	1.84 [1.14;2.97]*	1.72 [1.02;2.9]*	1.74 [0.95;3.20]
high HH income (ref. low HH income)	1.99 [1.29;3.07]**	1.69 [0.86;3.32]	3.6 [1.61;8.05]**	2.36 [0.95;5.85]
mixed neigh. (ref. Protestant neigh.)	0.85 [0.33;2.23]	1.04 [0.28;3.83]	—	—
Catholic neigh. (ref. Protestant neigh.)	0.81 [0.63;1.03]	0.83 [0.57;1.23]	0.59 [0.34;1.02]	0.65 [0.39;1.08]
no. post-boxes	1.08 [0.99;1.18]	1.10 [0.96;1.25]	1.30 [1.05;1.61]*	1.05 [0.86;1.28]
pop. density	1.00 [1.00;1.01]	1.00 [0.99;1.01]	1.02 [1.00;1.03]	1.02 [1.00;1.04]*
religious heterogeneity	1.00 [0.97;1.03]	1.00 [0.96;1.04]	1.09 [0.99;1.2]	1.02 [0.94;1.11]

In contrast, we found that SES best explained the variation in overall cooperative behaviour. At the mean values for all other traits, individuals in the highest income group were 25% more likely to donate than individuals in the lowest income group (table 2) and letters dropped in least deprived neighbourhoods had a 72% probability of being returned compared with 48% in the most deprived neighbourhoods (table 3). In relation to education, individuals with a university degree had an 80% probability of donating compared with 60% for individuals with only primary schooling (table 1). When looking at the specific types of cooperative behaviour, we found wealthy people and wealthy neighbourhoods associated with more help to the in-group and higher-educated people more likely to donate to the out-group and to Save the Children (tables 2 and 3). We also found that people with children were more likely to donate, but specifically to in-group not out-group schools (table 2).

## 4. Discussion

Our results indicate that while in a situation of intergroup conflict, individuals are more likely to reduce cooperation with out-group members, this will have no effect on cooperative behaviour towards the in-group. Current theoretical models of parochial altruism build on the assumption that increased pro-sociality or in-group altruism results in a group advantage in a situation of intergroup conflict [2–4]. Laboratory-based empirical results supporting these models are based on a game pay-off structure in which altruistic groups always outcompete selfish groups in a situation of group conflict [5,10]. Here, we question whether this assumption is realistic and argue that it is not generalizable to all situations where groups are in competition or conflict. In the case of Catholics and Protestants in Northern Ireland recent conflict between the two groups has mostly been over issues related to schools, housing and symbolic displays [21]; it is possible that in these situations increased group cohesion does not provide a group advantage, or that the individual cost of helping the group outweighs the potential group advantage.

In situations of intergroup conflict where within-group cooperation may provide a group advantage—such as intergroup warfare—the individual bearing the costs for the group may not be acting out of altruistic concerns. Instead the behaviour may be the result of reputation considerations [26], enforcement by other group members or the prospect of personal material gain [27], or may represent hierarchical dominance structures (e.g. conscription) in larger societies which may operate for the benefit of powerful individuals [28].

Our results point to the importance of SES in explaining the variation in cooperative behaviour as found in previous studies [29,30], and put in question the findings of previous studies on intergroup conflict and cooperation that fail to take into account the variation of individual SES [5,6,8–10]. The lower levels of cooperation found for individuals of low SES in behaviours where there is a monetary stake, such as the donations experiment, could be explained by the relative cost of cooperation being higher for poor individuals than for wealthy ones. The fact that we also find individuals in deprived neighbourhoods less likely to engage in an cooperative act without an associated monetary cost (i.e. posting a letter) is more surprising, but the harsh environment and possible shorter-time horizons of individuals experiencing income deprivation [31] may minimize the potential for long-term reciprocity leading to a general reduction in cooperative behaviour [32]. Notably, low-income individuals in our sample are less likely to trust people in their neighbourhoods lends support to this proximate explanation (see the electronic supplementary material).

Another possibility, proposed by Gavrilets & Fortunato [33], is that within-group inequality is driving the differential investment in the between-group conflict, with the different individual costs and benefits of intergroup conflict resulting in higher in-group contributions by high-status individuals. In other words, in a situation of intergroup conflict, high status individuals have more to gain or lose, and as a result, are more likely to invest in the in-group. According to this model, the behaviour of high-status individuals is seemingly altruistic at the within-group level, however, these individuals' behaviour is not motivated by altruism but rather by competition with their high ranking peers in other groups. Our results provide some empirical support for this model as we find that both wealthy individuals and neighbourhoods are more likely to contribute to the in-group, possibly indicating that wealthy Catholics and Protestant are more willing to invest in intergroup competition.

The fact that individuals with children were more likely to donate to an in-group, but not an out-group school, led us initially to assume that people wanted to benefit their own children's school. However, when re-analysing the data using instead the binary variable of children currently living at home (more likely to reflect children attending the nearby in-group school, than offspring who may have left the household) no significant effect was found (see the electronic supplementary material, table S6). This suggests that shared kinship is not the mediating mechanism for increased donations. People with children are also more likely to donate to the neutral charity Save the Children, but this might be related to a priming effect of people with children being more inclined to donate to a charity invoking children. It is also interesting to note that levels of neighbourhood religious heterogeneity do not affect cooperation, challenging the notion that group diversity undermines social cohesion [34].

There are some potential limitations to our study. It is possible that our neutral institutions were, in fact, perceived as biased towards one or other religious group, but neither religious background or threat levels significantly explain the variation in neutral donations or letters return. This suggests that neutral institutions are not particularly affiliated with either group. The donation experiment induces a possible priming effect on the participants, as it was conducted after the questionnaire. We decided against conducting the experiment before, as this might have raised suspicion from the participants that they were taking part in an experiment. We would expect a prime to amplify the effects of conflict on parochial altruism (i.e. increase in-group altruism and reduce out-group altruism), so the reduction in out-group cooperation might be less striking without priming. However, the prime should also enhance in-group cooperation if parochial altruism is operating, and as exposure to conflict in our primed experiment did not predict variation in in-group cooperation, the effect is not likely to be present without the prime either.

Our experimental design also does not allow us to resolve endogeneity issues, as the levels of exposure to violence may not be exogenous to individual cooperative behaviour; for example, younger, poorer and less-educated individuals may be more likely to be involved in sectarian conflict and as a result feel more threatened. In order to attenuate these endogeneity issues we control in our analysis for the contextual variables significantly correlated with intergroup conflict. Furthermore, we should be aware of the selection bias inherent to studies involving active participation, such as our donations experiment, as people willing to participate in the survey could be more cooperative than the wider population (i.e. participating in the study in itself may be a cooperative act) [13]. Nevertheless, we find our sample to be representative of the population at the neighbourhood, city and country level when comparing it with the 2011 UK Census data on of gender, religion, age, education and employment status (see the electronic supplementary material, table S3).

Finally, the lost letter experiment—which does not suffer from selection bias—largely replicates the donations' results, with both pointing to the importance of SES and how conflict negatively affects cooperative behaviour towards the out-group. Our results highlight the importance of empirically testing theoretical models by measuring large-scale cooperation in a real-world setting, and demonstrate how adversity, either from conflict or deprivation, leads to the breakdown of all types of cooperation.

## Supplementary Material

Supplementary Material

## Data accessibility

The dataset with survey, donations and lost letter returns information is available from Dryad, doi: dx.doi.org/10.5061/dryad.g722q.

## References

[1] Atkin R 1990 Pillar of fire: Dunkirk 1940. London, UK: Sidgwick & Jackson.

[2] Bowles S Choi J-K Hopfensitz A 2003 The co-evolution of individual behaviors and social institutions. J. Theor. Biol. 223, 135–147. (10.1016/S0022-5193(03)00060-2) 12814597

[3] Choi J-K Bowles S 2007 The coevolution of parochial altruism and war. Science 318, 636–640. (10.1126/science.1144237) 17962562

[4] García J van den Bergh JCJM 2011 Evolution of parochial altruism by multilevel selection. Evol. Hum. Behav. 32, 277–287. (10.1016/j.evolhumbehav.2010.07.007)

[5] Bornstein G 2003 Intergroup conflict: individual, group, and collective interests. Pers. Soc. Psychol. Rev. 7, 129–245. (10.1207/S15327957PSPR0702_129-145) 12676644

[6] Bernhard H Fischbacher U Fehr E 2006 Parochial altruism in humans. Nature 442, 912–915. (10.1038/nature04981) 16929297

[7] Voors M Nillesen E Verwimp P Bulte E Lensink R van Soest D 2012 Violent conflict and behavior: a field experiment in Burundi. Am. Econ. Rev. 102, 941–964. (10.1257/aer.102.2.941)

[8] Gneezy A Fessler DMT 2011 Conflict, sticks and carrots: war increases prosocial punishments and rewards. Proc. R. Soc. B 279, 219–223. (10.1098/rspb.2011.0805) PMC3223676 21653590

[9] Bauer M Cassar A Chytilova J Henrich J 2014 War's enduring effects on the development of egalitarian motivations and in-group biases. Psychol. Sci. 21, 47–57. (10.1177/0956797613493444) 24220626

[10] Puurtinen M Mappes T 2009 Between-group competition and human cooperation. Proc. R. Soc. B 276, 355–360. (10.1098/rspb.2008.1060) PMC2581672 18826935

[11] Arrow H 2007 The sharp end of altruism. Science 318, 581–582. (10.1126/science.1150316) 17962546

[12] Laury SK Taylor LO 2006 Altruism spillovers: are behaviors in context-free experiments predictive of altruism toward a naturally occurring public good? J. Econ. Behav. Organ. 65, 9–29. (10.1016/j.jebo.2005.05.011)

[13] Levitt SD List JA 2007 What do laboratory experiments measuring social preferences reveal about the real world? J. Econ. Perspect. 21, 153–174. (10.1257/jep.21.2.153)

[14] List JA 2007 On the interpretation of giving in dictator games. J. Polit. Econ. 115, 482–493. (10.1086/519249)

[15] Binmore K 2010 Social norms or social preferences? Mind Soc. 9, 139–157. (10.1007/s11299-010-0073-2)

[16] Henrich J 2005 ‘Economic man’ in cross-cultural perspective: behavioral experiments in 15 small-scale societies. Behav. Brain Sci. 28, 795–815; discussion 815–855 (10.1017/S0140525X05000142) 16372952

[17] Burton-Chellew MN West SA 2013 Prosocial preferences do not explain human cooperation in public-goods games. Proc. Natl Acad. Sci. USA 110, 216–221. (10.1073/pnas.1210960110) 23248298 PMC3538240

[18] House BR Silk JB Henrich J Barrett HC Scelza BA Boyette AH Hewlett BS McElreath R Laurence S 2013 Ontogeny of prosocial behavior across diverse societies. Proc. Natl Acad. Sci. USA 110, 14 586–14 591. (10.1073/pnas.1221217110) PMC3767518 23959869

[19] BBC NEWS. 2001 Dispute school extra cash criticised.

[20] Sutton M 2012 Sutton index of deaths. Sutt. Index Deaths. See http://cain.ulst.ac.uk/sutton/.

[21] Nolan P 2012 Northern Ireland peace monitoring report. Community Relations Council (no. 1).

[22] Northern Ireland Life & Times Survey. 2005 Marriage partner: religion. North. Irel. LIFE TIMES Surv. See http://www.ark.ac.uk/nilt/2005/.

[23] Milgram S Mann L Harter S 1965 The lost-letter technique: a tool of social research. Pub. Opin. Q. 29, 437 (10.1086/267344)

[24] Kahneman D Knetsch J Thaler RH 1986 Fairness and the assumptions of economics. J. Bus. 59, S285 (10.1086/296367)

[25] Snijders TAB Bosker RJ 2011 Multilevel analysis: an introduction to basic and advanced multilevel modeling. 2nd edn London, UK: Sage.

[26] Nowak MA Sigmund K 1998 The dynamics of indirect reciprocity. J. Theor. Biol. 194, 561–574. (10.1006/jtbi.1998.0775) 9790830

[27] Mathew S Boyd R 2011 Punishment sustains large-scale cooperation in prestate warfare. Proc. Natl Acad. Sci. USA 108, 11 375–11 380. (10.1073/pnas.1105604108) PMC3136302 21670285

[28] Guala F 2012 Reciprocity: weak or strong? What punishment experiments do (and do not) demonstrate. Behav. Brain Sci. 35, 1–15. (10.1017/S0140525X11000069) 22289303

[29] Wilson DS O'Brien DT Sesma A 2009 Human prosociality from an evolutionary perspective: variation and correlations at a city-wide scale. Evol. Hum. Behav. 30, 190–200. (10.1016/j.evolhumbehav.2008.12.002)

[30] Holland J Silva AS Mace R 2012 Lost letter measure of variation in altruistic behaviour in 20 neighbourhoods. PLoS ONE 7, e43294 (10.1371/journal.pone.0043294) 22905250 PMC3419711

[31] Nettle D 2010 Dying young and living fast: variation in life history across English neighborhoods. Behav. Ecol. 21, 387–395. (10.1093/beheco/arp202)

[32] Barta Z McNamara JM Huszár DB Taborsky M 2011 Cooperation among non-relatives evolves by state-dependent generalized reciprocity. Proc. R. Soc. B 278, 843–848. (10.1098/rspb.2010.1634) PMC3049049 20861047

[33] Gavrilets S Fortunato L 2014 A solution to the collective action problem in between-group conflict with within-group inequality. Nat. Commun. 5, 1–11. (10.1038/ncomms4526) PMC3974216 24667443

[34] Alesina A La Ferrara E 2005 Ethnic diversity and economic performance. J. Econ. Lit. 43, 762–800. (10.1257/002205105774431243)

