# Supplementary material for: Cooperation and conflict: field experiments in Northern Ireland
Source: Proc Biol Sci. 2014 Oct 7;281(1792):20141435. doi: 10.1098/rspb.2014.1435 (PMC4150329; doi:10.1098/rspb.2014.1435)
Supplement: Supplementary Material [file rspb20141435supp1.pdf]

Title: Cooperation and Conflict: Field Experiments in Northern Ireland

Authors: Antonio S. Silva[1]*, Ruth Mace[1]

Affiliations:

[1]Department of Anthropology, University College London, UK

*Correspondence to: antonio.silva.09@ucl.ac.uk

Supplementary Material

Table of Contents:

1. Study Site 1
2. Descriptive Statistics 3
3. Materials and Methods 7
4. Results 13
5. References 17

1. Study Site

Belfast is the capital and main urban area of Northern Ireland – a region of the United Kingdom - with a population of over 500.000 and approximately an equal split of Catholic and Protestant composition [1] (figure S1). The levels of residential segregation of these two populations are high, with two thirds of the population living in areas made up of over 80% of their own religious group [2] and 93% of public housing having more than 80% of one single group [3]. The segregation between these two communities in Northern Ireland extends beyond residential division and starts at an early age with a heavily segregated educational system. In 2010/11, 94% of nursery, primary and secondary schools were either predominantly Catholic (managed by the Catholic Church) or Protestant (managed by the state or Protestant Churches). The other 6% were integrated schools which actively promote mixed schooling, but have had limited success with little increase in the number of students enrolled in past years [4]. The majority of Protestant schools are managed by the state and are, in principle, not sectarian, but they are attended almost exclusively by children of Protestant origin; for example, in our sample only 2.2% of children from Catholic parents attend a Protestant school. The high levels of residential and educational segregation are also reflected in the low rates of inter-marriage, with only 12% marriages in 2005 being of

different religions [5]. This rate of inter-marriage has been slowly increasing over the past decade, but for the duration of the conflict it remained around 5% [6].

Belfast also presents a wide range of socio-economic conditions, with the richest and the poorest areas of the whole Northern Ireland found here. At the country level, this distribution of wealth is also associated with religious affiliation, with the Catholic community being historically more deprived than the Protestant community. At the present, 26% of Catholics live in low income households, compared with only 16% of Protestants [4]. The situation in Belfast mirrors this situation, with the majority of deprived neighbourhoods being predominantly Catholic [7].

The sectarian conflict in Northern Ireland is still prevalent, although with lower intensity and frequency than in past. The Good Friday agreement in 1997 and the parallel economic development of the region have reduced the levels of violence in the past decade. However riots in deprived neighbourhoods in Belfast are still frequent, especially at the Orange March Parades (Protestant community groups) during the summer months. In September, 2012 riots over 3 consecutive days in north Belfast resulted in the injury of 60 police officers and the arrest of over 30 people [8]. In 2011 alone, there were 64 sectarian bombings, 60 sectarian shootings and in 2010/11, 995 sectarian related crimes were recorded, ranging from fights and church attacks to homicide [4].

Figure S1. Neighbourhood religious composition of Belfast, Northern Ireland: 0%-20% Catholic (Red), 21%-40% (Orange), 41%-60% (Yellow), 61%-80% (Light Green), 81%-100% (Green)

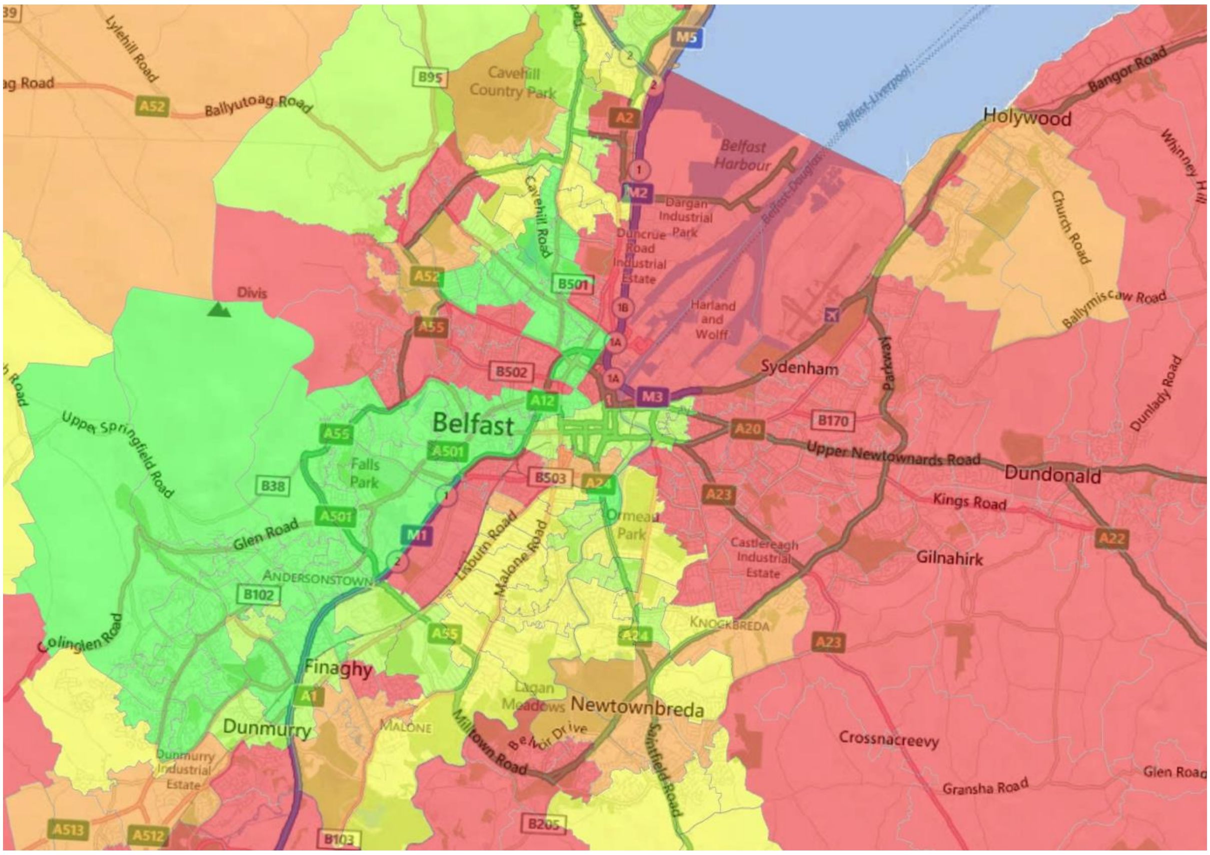

2. Descriptive Statistics

Donations

Participants in our study were able to choose how much money they wanted to donate to a charity or school, but 94% of the participants chose to either keep or donate the total amount (table 1). As a result, we decided to use a logistic regression with a binary response variable of the participant choosing to donate any amount or to keep it all.

Explanatory Variables - Individual Level (table S1)

Education: Ordinal variable based on questionnaire response on the highest educational level achieved.

Gender: Nominal variable of gender.

Household income: Ordinal variable of the terciles of household income in pounds equivalised using the OECD modified scale to adjust for household size and composition [9].

Religion: Binary variable based on questionnaire response to the religion the individual was brought up. The various denominations of Protestant religion were aggregated to Protestant and individuals from other religions and with no religion were excluded from the analysis

Children: Binary variable based on questionnaire response to how many children the individual had.

Sectarian Threat Index: Continuous variable from a polychoric factor analysis on six categorical variables (table S2) based on individual questionnaire responses described below (mean=1.8; s.d.=0.7)

Table S1. Percentage distribution of individual variables used in the analyses of donations (Sample B)

VARIABLE	%
**Educational Level**	
Primary School	27.5
GCSE	28.3
A-Level	18.5
Undergraduate	15.9
Graduate	9.9
**Gender**	
Female	53.9
Male	46.1
**Household Income**	
1st Tercile	33.5
2nd Tercile	38.2
3rd Tercile	28.3
**Religious Background**	
Catholic	53.2
Protestant	46.8
**Children**	
No children	29.2
One or more	70.8

Table S2 Percentage distribution, factor loadings and unique variances of variables that are part of the factor variable sectarian threat index

**SECTARIAN THREAT INDEX**		
**Variable**		
**Uncomfortable in different neighbourhood**	**Factor loading**	**Uniqueness**
	0.4936	0.7563
**Response**	**Percentage**	
Yes	47.2	
No	51.7	
Not sure	1.1	
**Community under threat**	**Factor loading**	**Uniqueness**
	0.6894	0.5248
**Response**	**Percentage**	
Yes	15	
No	84.1	
Not sure	0.9	
**Reduced Segregation**	**Factor loading**	**Uniqueness**
	0.6557	0.57
**Response**	**Percentage**	
Strongly agree	10.5	
Agree	41.9	
Neither agree nor disagree	10.1	
Disagree	27.9	
Strongly disagree	9.7	
**Sectarian Attack**	**Factor loading**	**Uniqueness**
	0.5936	0.6476
**Response**	**Percentage**	
No	87.3	
Rarely	3.2	
A few times	6.9	
Many times	2.6	
**Neighbourhood violence**	**Factor loading**	**Uniqueness**
	0.8206	0.3266
**Response**	**Percentage**	
Not often a problem	70.6	
Sometimes a problem	21.7	
Often a problem	3.9	
Very often a problem	3.9	
**Neighbourhood discrimination**	**Factor loading**	**Uniqueness**
	0.796	0.3664
**Response**	**Percentage**	
Not often a problem	73.6	
Sometimes a problem	16.7	
Often a problem	4.9	
Very often a problem	4.7	

SURVEY QUESTIONS – Sectarian Threat Index

Community under threat
Do you feel that your community is currently under threat from others outside of it?

Reduced Segregation
Would you agree that the segregation between religious communities is less pronounced since the Good Friday agreement?

Sectarian Attack
In the past year have you been attacked, threatened or insulted because of your religious/political background?

Uncomfortable in different neighbourhood
Would you feel uncomfortable walking around in certain neighbourhoods because you feel people there are from a different religion to your own?

Neighbourhood violence

Please indicate how much of a concern you feel sectarian violence is in your neighbourhood

Neighbourhood discrimination

Please indicate how much of a concern you feel sectarian discrimination is in your neighbourhood

Explanatory Variables (neighbourhood level)

Religious Composition:
The religion composition was measured by the percentage of Catholic individuals living in a neighbourhood. This measure represents the number of people that were brought up as Catholic and originates from the UK Census 2011 [7]. This measure was added to the Northern Ireland part of the Census in 2001 due to the large percentage of people that refused to answer an alternative question in previous Census, in which respondents are asked for their individual religion instead of their community background (12% of no answer vs. 3% of no answer [10]). The high rate of non-answers in the original question may reflect various aspects, but is likely to be the result of the sensitive nature of religion in Northern Ireland. The mean Catholic composition in the sample of 22 neighbourhoods used is 50.0% (s.d.=34.6).

In order to simplify the analysis of the data, the neighbourhood religious composition was divided into three categories: predominantly Protestant (0% – 25% Catholic), mixed (25% - 75% Catholic) and predominantly Catholic (75% - 100% Catholic). This division has previously been used in other studies looking at religious segregation in Northern Ireland

[11,12] and it appears to capture the predominant characteristics of the neighbourhood's religious composition.

Religious Heterogeneity:

The religious heterogeneity of a neighbourhood is the absolute value of the subtraction of the neighbourhood religious composition by 50% (mixed neighbourhood). The mean religious heterogeneity in the sample of 22 neighbourhoods used is 28.8 (s.d. = 15.8) and ranges from 3.2 (heterogeneous neighbourhood) to 44.9 (homogeneous neighbourhood).

Household Income

This variable is the mean value in pounds of the individual values of household income at the neighbourhood level (n=891; mean=13937.5; s.d.= 16296.9).

Post-boxes:

The number of post-boxes in each neighbourhood was counted using Google Maps and post box location data from Somerville [13]. This variable was used as a control in the analysis as it was hypothesised that letters were more likely to be returned in neighbourhoods with a higher number of post-boxes. There were an average of 3.1 post-boxes per neighbourhood (s.d. = 1.5).

Population Density:

This measure was obtained from the UK Census 2011 [7] and indicates the number of people per hectare that live in a neighbourhood. This was used in the analysis as a control variable as it was hypothesised that densely populated neighbourhoods have more people passing by the lost letters and as a result are more likely to pick them up. In the sampled neighbourhoods there were an average of 43.1 individuals per hectare (s.d. = 21.8).

Sectarian Threat Index (neighbourhood level):

This variable is the mean value of the individual values of sectarian threat index at the neighbourhood level (n=941; mean=1.6; s.d.= 0.4).

3. Materials and Methods

<u>Sampling Methods</u>

The survey and the lost letter experiment were conducted in 22 different Belfast neighbourhoods (n=948; **Sample A**), and the donation experiment was conducted in a 16

neighbourhoods sub-sample (n=497; **Sample B**) of the original 22 (Fig. S2). These were selected to include a wide range of variation in income deprivation and religious composition. Each neighbourhood represents a lower super output area (LSOA), which is a UK standard geographic unit with an average population of 1800 (ranging from 1300 to 2800) providing the smallest area where extensive contextual socio-economic data is available [14]. There are a total of 890 LSOAs in Northern Ireland which were generated taking into account "population size, mutual proximity and social homogeneity" [14].

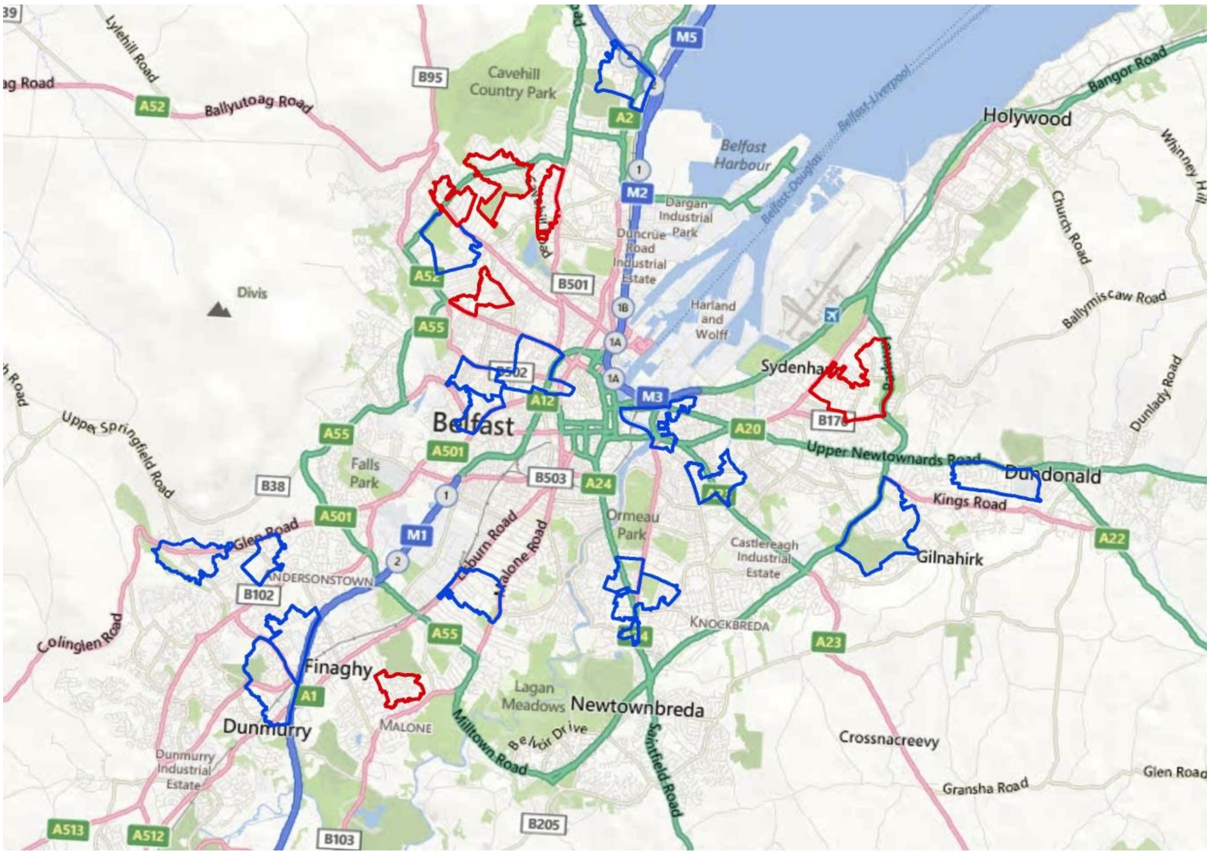

Figure S2. Neighbourhood sample of 22 (Sample A; Blue and Red) and 16 (Sample B; Blue) neighbourhoods.

The selection process of these neighbourhoods was carefully considered in order to be able to provide enough variation to test the relevant hypotheses. As a result, the selection attempted to include neighbourhoods with various conditions of sectarian conflict, socio-economic characteristics and religious composition. We are aware of the bias inherent to this selection process, but a random sample of neighbourhoods would be unlikely to have provided sufficient variation to test the relevant hypotheses of this study.

In each neighbourhood, the survey and donations sampling was conducted by going to every house in the neighbourhood from 10 am to 8pm, with a visit at the weekend for each of the neighbourhoods. The robustness of the sample was verified by comparing the representativeness of the sample with the 2011 UK Census data on of gender, religion, age, education and employment status, which shows our sample to be representative of the population at the neighbourhood, city and country level (table S3).

Table S3. Frequency distribution of gender, religion, age, highest educational level achieved and employment status of the individuals in Sample A (n=948), Sample B (n=497), and the census data for the same neighbourhoods in the two samples, in Belfast and in Northern Ireland. * - the unemployed data for the Census corresponds to unemployed individuals of active age, disabled or sick and their full-time carers.

	Sample A	Sample B	Census 2011 (Sample A)	Census 2011 (Sample B)	Census 2011 (Belfast)	Census 2011 (NI)
**Male**	46.8	46.1	47.6	47.5	48.1	49.2
**Female**	53.2	53.9	52.4	52.5	51.9	50.8
**Catholic**	50.0	53.2	51.0	54.3	48.6	44.6
**Protestant**	50.0	46.8	42.0	38.6	42.3	49.5
**Mean Age**	45.2	45.1	37.0	37.8	37.0	37.6
**Education**						
**Primary School**	23.7	27.5	N/A	N/A	N/A	N/A
**GCSE**	29.5	28.3	25.2	25.1	23.3	26.9
**A-Level**	18.9	18.5	10.8	10.5	13.4	12.1
**Undergraduate**	17.6	15.9	25.5	25.8	26	22.7
**Graduate**	10.3	9.9				
**Employment**						
**Unemployed***	25.1	24.7	23.9	24.2	23.2	19.6
**Employed FT**	39.6	40.3	40.4	40.8	39.7	44.5
**Employed PT**	10.1	9.9	13.8	13.4	12.6	13.1
**Student FT**	5.4	4.5	6.1	6.0	7.4	6.2
**Student PT**	0.2	0.2				
**Retired**	19.6	20.4	12.0	11.8	11.6	12.9

Survey

The survey was completed in person by three trained assistants at the respondents' house during May and June 2012 (n=948) in 22 Belfast neighbourhoods. The survey was conducted between 10.00 and 20.00 during weekdays and weekends. Each assistant was allocated a set

of streets in the neighbourhood and then knocked on people's door asking if they would like to take part in the questionnaire. The questionnaire consisted of 50 questions, required about 10 minutes to complete and was structured with multiple-choice responses that the researcher read out and for which the respondent chose the most appropriate choice.

The questionnaire addressed a range of issues with a focus on questions about the respondents' socio-economic characteristics (age, sex, religion, employment status, education, income), religious beliefs (frequency of attendance of religious services and the importance of god and religious values in the daily life), attitudes towards the out-group (attitudes on inter-group marriage and educational segregation), levels of interaction with kin (distance and frequency of contact with kin) and inter-group contact (number of friends from different religious group) and perception of neighbourhood's social cohesion.

Donations

The experiment was conducted after the completion of the questionnaire with a random sub-sample of 497 individuals taking part in the survey in 16 neighbourhoods (32 individuals were excluded from the analyses due to to missing data on household income and religious background). The participants were informed that they would receive a £5 financial incentive for completing the questionnaire and were given the possibility to donate part of that money to a local primary school (Protestant or Catholic) or charity (Save the Children). Individuals were only offered the option to donate to a single institution, which was randomly allocated. The selection of the primary schools was conducted by choosing the nearest Catholic and Protestant school to the centroid of the neighbourhood using Google Maps.

After the completion of the questionnaire, the researcher hands the participant the financial incentive in the form of 5 pound coins, followed by informing the participant that they are welcome to keep the money or if they prefer they can donate part or all of the money to a local school or charity. At the same time, the researcher presents, in view of the participant, a charity box with the name of the local school or charity (figure S3), where he or she can drop some or all of the coins. There were three treatments - one for each of the local schools and one for the charity – and participants were only given the choice to donate to one of the three options. The amount donated to the local school treatments measures in-group (if participant is of the same religion as the school) and out-group cooperation (if participant is of different

religion as the school), and the charity treatment measures unbiased cooperation. There was no significant effect of the assistant on the amount of donations (GLM, $p>0.05$).

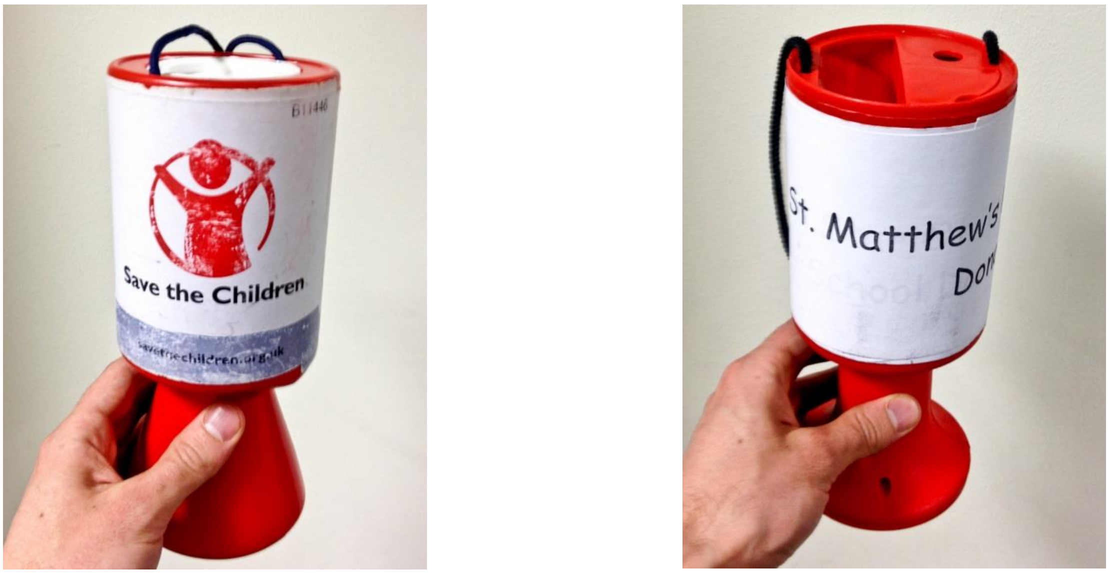

Figure S3. Donation boxes: Save the Children (left) and Catholic Primary school (right)

<u>Lost Letters</u>

The lost letter experiment was conducted in the sample of 22 neighbourhoods and a total of 1056 lost letters were dropped in two rounds in May and June 2012 (n=792) and 2013 (n=264). The final analysis used a restricted sample of 832 letters that included biased letters dropped only on predominantly Catholic and Protestant neighbourhoods (>75% composition) resulting in a final sample of 352 neutral letters in 22 neighbourhoods, and 240 in-group letters and 240 out-group letters in 15 neighbourhoods. Overall, letters dropped in 2013 were less likely to be returned (63.9% in 2012; 53.9% in 2013; GLM: OR=0.66, $p<0.01$), but there was no significant year effect on the differential return of the different type of letters.

These stamped letters were addressed to either fictional sectarian or neutral charities (CatholicAID, ProtestantAID and CancerAID; figure S4) and were dropped by two researchers on the pavement with the address facing up on rain free days. To avoid a return bias dependant on the day and time that the letters were dropped (e.g. when the postman or street cleaners come), each neighbourhood was visited three times at three different time slots (morning, lunchtime and afternoon) on three different days. The letter drop points in the neighbourhood were randomly determined using ArcGIS 10. The return rates of the letters from the three treatments (Catholic, Protestant and neutral) were used to measure the neighbourhood levels of in-group, out-group and unbiased cooperation.

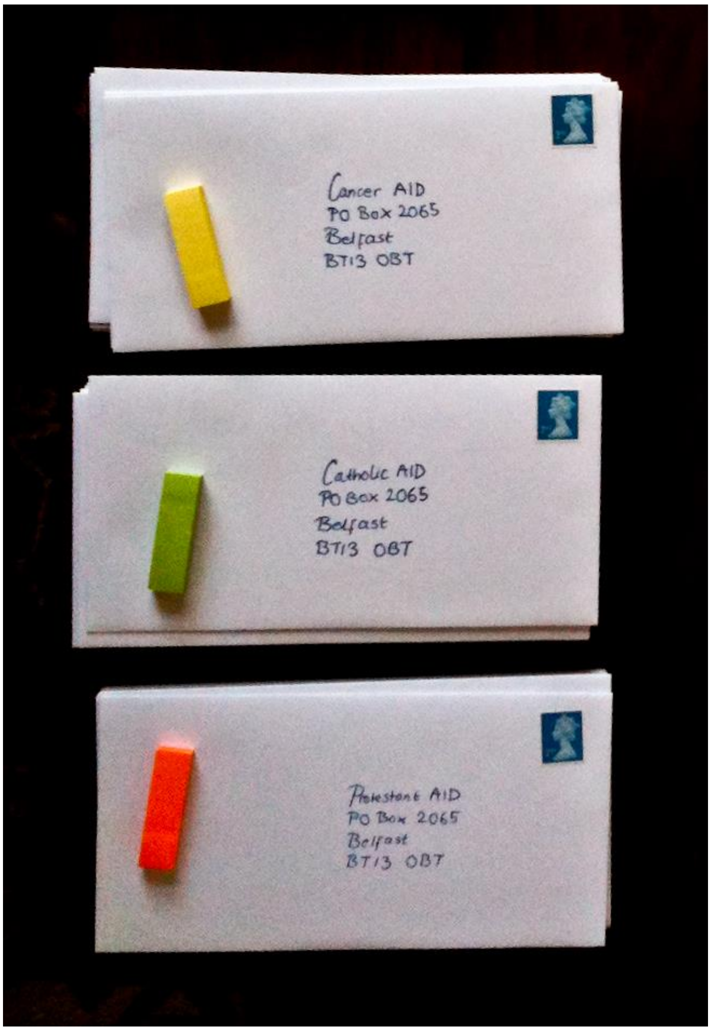

Figure S4. Lost letters: CancerAID (top), CatholicAID (middle) and ProtestantAID (bottom).

4. Results

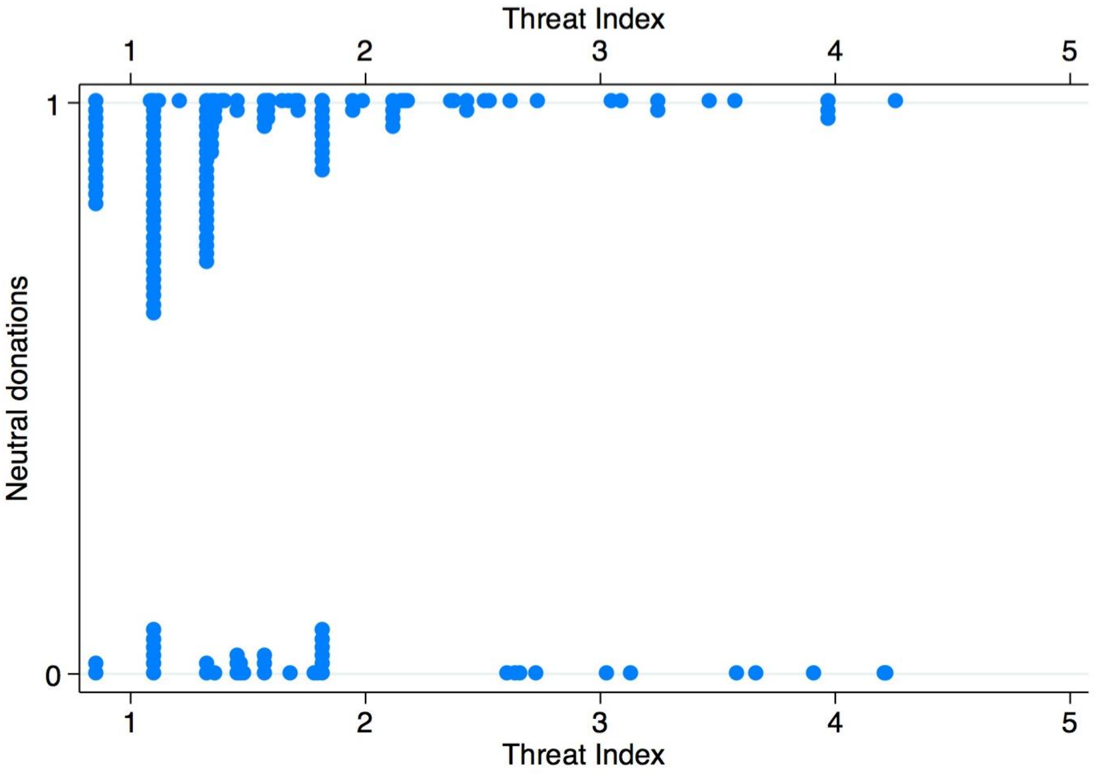

Figure S5. Dot-plot of raw data of donations to Save the Children by the individual threat index. Height of dots represents number of tied donations by the same value of threat index (n=158)

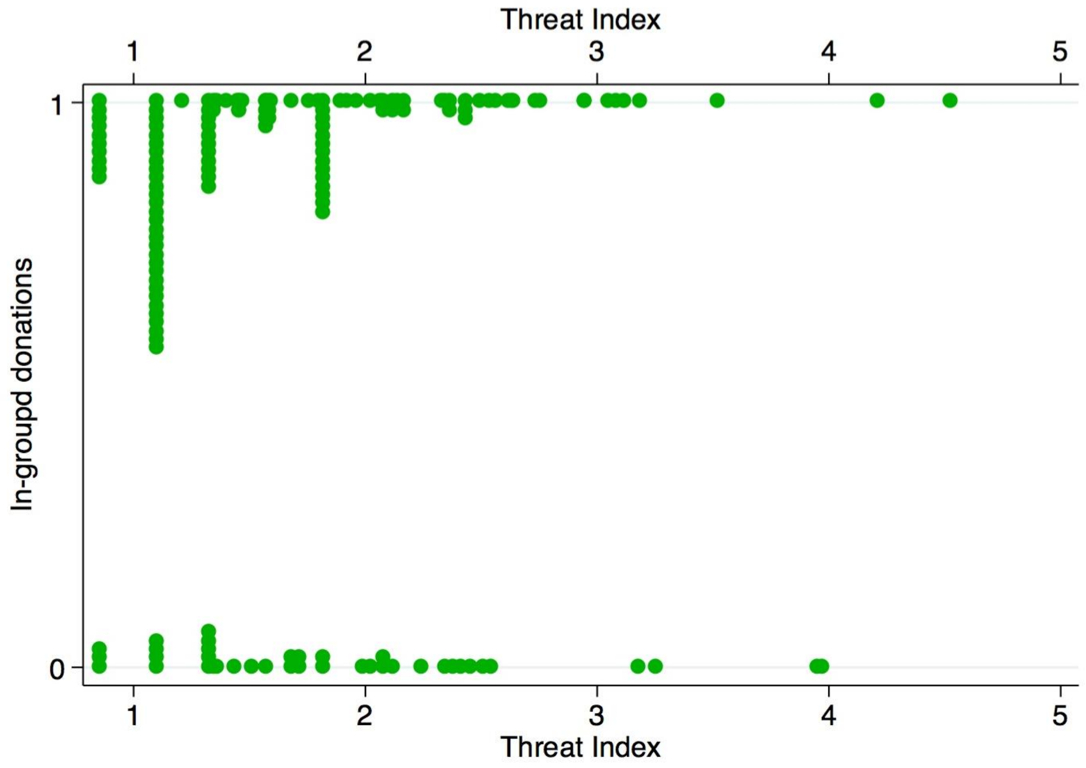

Figure S6. Dot-plot of raw data of donations to an in-group school by the individual threat index. Height of dots represents number of tied donations by the same value of threat index (n=153)

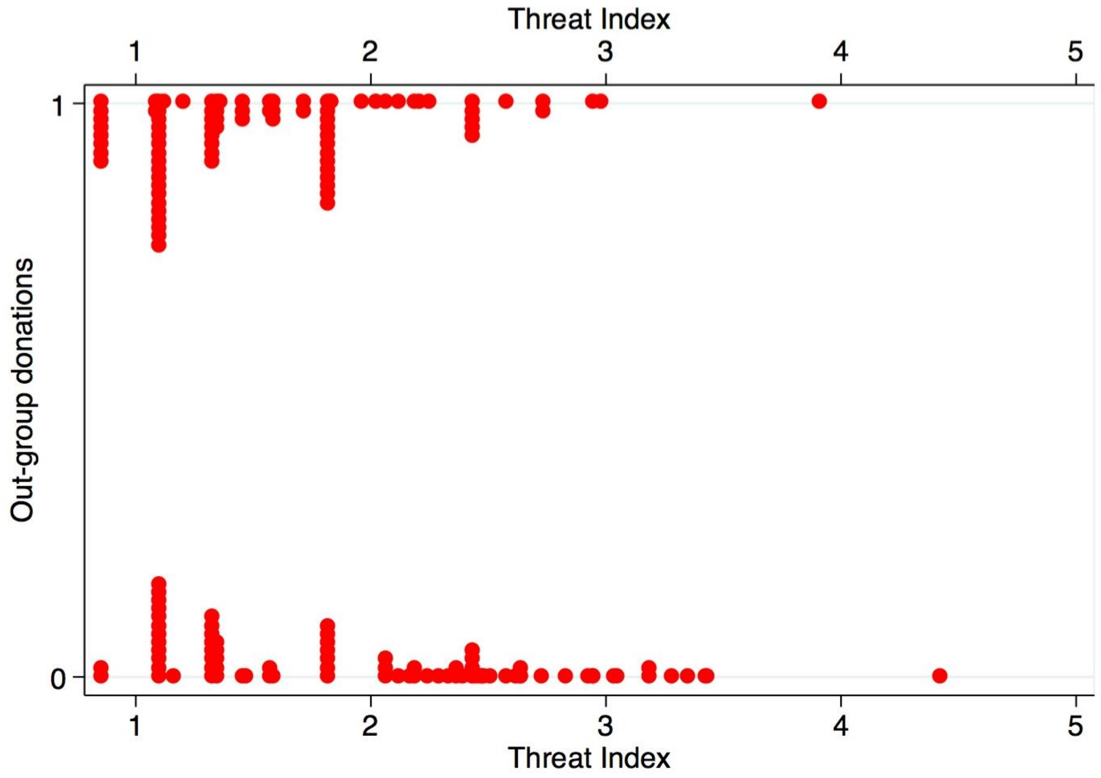

Figure S7. Dot-plot of raw data of donations to an out-group school by the individual threat index. Height of dots represents number of tied donations by the same value of threat index (n=155)

Parochialism

Table S4. Incidence rate ratios, 95% confidence intervals and predicted probabilities from a multi-level logistic regression used to predict donations to neutral, in-group and out-group institutions (Save the Children, Catholic or Protestant primary schools)

Type of Donation	IRR [95 CI]
**Neutral (ref. out-group)**	3.01 [1.89;4.81] ***
**In-group (ref. out-group)**	2.99 [1.87;4.79] ***
	**Pred. Probability**
**Neutral**	0.76 [0.7;0.83]
**In-group**	0.76 [0.7;0.83]
**Out-group**	0.51 [0.44;0.59]

***p<.001; **p<.01; *p<.0.05; .p<0.1

Table S5. Incidence rate ratios, 95% confidence intervals and predicted probabilities from a logistic regression used to predict the return of neutral, in-group and out-group lost letters

Type of Letter	IRR [95 CI]
**Neutral (ref. out-group)**	1.99 [1.5;2.64] ***
**In-group (ref. out-group)**	1.57 [1.16;2.12] **
	**Pred. Probability**
**Neutral**	0.67 [0.63;0.71]
**In-group**	0.62 [0.57;0.67]
**Out-group**	0.51 [0.45;0.56]

***p<.001; **p<.01; *p<.0.05; .p<0.1

Table S6. Incidence rate ratios and 95% confidence intervals from multi-level logistic regressions used to predict overall donations (n=466), and donations to neutral (n=158), in-group (n=153) and out-group institutions (n=155) (Save the Children, Catholic or Protestant primary schools). In the these models the binary variable of having children has been replaced by the binary variable of having children in the household.

**donations**	**overall**	**neutral**	**in-group**	**out-group**
**variable**	**IRR [95 CI]**	**IRR [95 CI]**	**IRR [95 CI]**	**IRR [95 CI]**
**Out-group donation** (ref. in-group)	1.17 [0.48;2.86]	-	-	-
**Neutral donation** (ref. in-group)	1.34 [0.62;2.93]			
**Threat Index**	1.1 [0.8;1.52]	0.94 [0.69;1.28]	1.11 [0.76;1.62]	0.62 [0.39;0.99]
**Threat Index X Out-group donation** (ref. Threat Index X In-group donation)	0.58 [0.35;0.95]	-	-	-
**Threat Index X Neutral donation** (ref. Threat Index X In-group donation)	0.82 [0.54;1.24]	-	-	-
**GCSE** (ref. primary school)	1.29 [0.89;1.87]	1.38 [0.74;2.58]	1.16 [0.61;2.23]	1.99 [0.9;4.42]
**A-Level** (ref. primary school)	1.50 [0.97;2.31]	1.22 [0.63;2.36]	1.46 [0.55;3.88]	2.96 [1.2;7.32]
**Undergraduate** (ref. primary school)	1.27 [0.80;2.03]	2.10 [0.91;4.86]	0.87 [0.38;1.98]	1.89 [0.67;5.34]
**Graduate** (ref. primary school)	1.84 [1.02;3.30]	1.43 [0.53;3.84]	1.15 [0.35;3.82]	6.17 [1.83;20.77]
**Mid HH Income** (ref. low HH income)	1.38 [0.99;1.93]	1.63 [0.84;3.15]	1.43 [0.80;2.57]	1.27 [0.66;2.44]
**High HH Income** (ref. low HH income)	1.78 [1.19;2.65]	1.63 [0.84;3.15]	3.06 [1.35;6.92]	1.14 [0.50;2.62]
**Male** (ref. female)	1.06 [0.82;1.38]	1.11 [0.69;1.81]	1.35 [0.85;2.15]	0.76 [0.45;1.30]
**Age**	1.01 [1.01;1.02]	1.02 [1.00;1.03]	1.03 [1.01;1.05]	1.01 [0.99;1.02]
**Protestant** (ref. Catholic)	0.78 [0.59;1.03]	0.75 [0.47;1.19]	1.01 [0.63;1.62]	0.69 [0.4;1.17]
**Children** (ref. no children)	1.24 [0.95;1.62]	1.46 [0.90;2.37]	1.06 [0.67;1.67]	1.16 [0.69;1.94]
**Religious Heterogeneity**	1 00 [1.00;1.01]	1.00 [0.99;1.02]	1.01 [0.99;1.03]	1 .00 [0.98;1.01]

**p<.01; *p<.0.05; .p<0.1

Trust and Income Deprivation

In order to further understand how associations between income deprivation and lower levels of cooperative behaviour are mediated, we ran a univariate zero inflated Poisson model to test whether household income was associated with levels of individual trust in their neighbourhood. We find that the wealthier individuals are less likely to distrust their neighbours than more deprived individuals; individuals in the highest income tercile are significantly less distrusting than individuals in the lowest income tercile (-0.56 [-.88; -.24]; p = 0.001)

Neighbourhood Trust is an ordinal variable based on questionnaire question "*How many people in your neighbourhood do you feel can be trusted?"*. The response distribution is: *Most* – 59.9%; *Some* – 26.7%; *Hardly Any* – 9.6%; *None* = 3.8%

5. References

1. Equality Comission for Northern Ireland 2006 Community background in Northern Ireland.

2. Shirlow, P. & Murtagh, B. 2006 *Belfast: Segregation, violence and the city*. London: Pluto Press.

3. Byrne, J., Hansson, U. & Bell, J. 2006 Mixed residential communities in Northern Ireland.

4. Nolan, P. 2012 Northern Ireland Peace Monitoring Report.

5. Northern Ireland Life & Times Survey 2005 Marriage Partner - Religion. *North. Irel. LIFE TIMES Surv.*

6. Moxon-Browne, E. 1991 National Identity in Northern Ireland. In *Social Attitudes in Northern Ireland: The First Report* (eds P. Stringer & G. Robinson),

7. NISRA 2012 Census 2011 - Population and Household Estimates for Northern Ireland.

8. BBC News 2012 Man is charged over riots in Denmark Street, Belfast.

9. Hagenaars, A. J. M., De Vos, K. & Zaidi, M. A. 1996 *Poverty statistics in the late 1980s: Research based on micro-data*. Office for Official Publications of the European Communities.

10. Osborne, B. 2002 Fascination of religion head count. *BBC News*.

11. Shuttleworth, I. G., Lloyd, C. D. & Martin, D. 2011 Exploring the implications of changing census output geographies for the measurement of residential segregation: the example of Northern Ireland 1991–2001. *J. R. Stat. Soc. Ser. A (Statistics Soc.* **174**, 1–16.

12. Shuttleworth, I. G. & Lloyd, C. D. 2009 Are Northern Ireland's communities dividing? Evidence from geographically consistent Census of Population data, 1971–2001. *Environ. Plan. A* **41**, 213–229.

13. Somerville, M. 2012 Find you nearest postbox.

14. ONS 2005 Super Output Areas (SOAs).
